# Socioeconomic differences in the cost-effectiveness of a telephone-based intervention for obesity prevention in early childhood

**DOI:** 10.1038/s41366-025-01904-4

**Published:** 2025-09-06

**Authors:** Thomas Lung, Alison Hayes, Li Ming Wen, Huilan Xu, Vicki Brown, Louise A. Baur, Philayrath Phongsavan, Anagha Killedar

**Affiliations:** 1 Sydney School of Public Health, Faculty of Medicine and Health, The University of Sydney, Sydney, NSW Australia; 2 The George Institute for Global Health, University of New South Wales, Sydney, NSW Australia; 3 Leeder Centre for Health Policy, Economics, and Data, Faculty of Medicine and Health, The University of Sydney, Sydney, NSW Australia; 4 Health Promotion Unit, Population Health Research & Evaluation Hub, Sydney Local Health District, Sydney, NSW Australia; 5 Sydney Institute for Women, Children and Their Families, Sydney Local Health District, Sydney, NSW Australia; 6 Deakin Health Economics, Institute for Health Transformation, School of Health and Social Development, Deakin University, Geelong, VIC Australia; 7 Sydney Medical School, The University of Sydney, Sydney, NSW Australia; 8 Prevention Research Collaboration, Sydney School of Public Health, Faculty of Medicine and Health, The University of Sydney, Sydney, NSW Australia; 9 Charles Perkins Centre, The University of Sydney, Sydney, NSW Australia

**Keywords:** Paediatrics, Translational research

## Abstract

**Objectives:**

This study investigated the cost-effectiveness of an early childhood obesity prevention intervention providing telephone and short message service (SMS) support to mothers of children aged 2–4 years by socioeconomic position (SEP).

**Methods:**

A model-based SEP-specific economic evaluation of the intervention was conducted. SEP-specific intervention costs and effects at age 5 years were derived from the trial data and applied to a cohort of 4- to 5-year-old Australian children. We used the validated EQ-EPOCH microsimulation model to predict SEP-specific body mass index (BMI) trajectories, quality-adjusted life years (QALYs) and health care costs until 17 years of age. Incremental cost-effectiveness ratios (ICERs) and acceptability curves were derived for each SEP group, using 2023 Australian dollars (AUD).

**Results:**

From an Australian health payer perspective, the ICERs for the low-SEP group were $131 per BMI unit avoided and $6549 per QALY gained, compared to the high-SEP group at $1161 per BMI unit avoided and $41,462 per QALY gained. Results were robust to sensitivity analyses varying the intervention effect size, intervention costs, healthcare costs, discount rate and disutility from overweight. The probability that the intervention was cost-effective at a willingness-to-pay threshold of $50,000 per QALY gained was extremely high in the low-SEP group (99.7%) and marginally cost-effective in the high-SEP group (49.6%).

**Conclusions:**

A telephone and SMS intervention was more cost-effective in low-SEP groups compared with high-SEP groups. Prioritizing families from socioeconomically disadvantaged backgrounds for this intervention will reduce healthy weight inequalities in childhood.

## Background

Early childhood obesity is a significant public health issue, with an estimated 37 million children aged under 5 years experiencing overweight or obesity worldwide in 2022 [1]. Childhood obesity is associated with poorer health [2, 3], increased healthcare costs [4, 5] and school absenteeism [6]. For Australian children, especially those of a lower socioeconomic position (SEP), overweight or obesity in early years frequently persists through childhood and into adolescence [7]. SEP in children is defined as a measure of the family and household’s social circumstances, including factors such as income, education level and occupation [8].

The first 2000 days of a child’s life is seen as a critical opportunity to establish healthy lifestyle behaviours in reducing the risk of obesity [1]. Recent national health surveys have shown socioeconomic inequality in the prevalence of childhood obesity, with children living in low socioeconomic areas twice as likely to have obesity as those living in high socioeconomic areas in Australia [3], and this inequality appears to be widening [9]. It is likely that social factors and opportunities, rather than biological inevitability, account for the variations in obesity prevalence, which should be viewed as an inequity. Recent systematic reviews of randomised controlled trials (RCTs) have shown some early childhood obesity prevention interventions are effective in improving behaviours such as sleep, diet and physical activity and reducing body mass index (BMI) [10, 11]. However, few studies reported effectiveness results that were stratified by SEP [12, 13].

Given the increasing strain on healthcare budgets, it is important that effective early childhood obesity interventions represent good value for money and are focused on improving equity. Economic evaluation compares the costs and outcomes of interventions to assist decision-making about how best to allocate health care resources [14]. Health economic modelling is important as it provides policymakers evidence of the longer-term impacts on health and healthcare costs associated with the intervention. To our knowledge, only two modelled economic evaluations have incorporated findings of SEP-specific change in BMI from RCTs in early childhood into their cost-effectiveness analyses [15, 16].

Cost-effectiveness may differ across SEP groups for several reasons. For example, effects of the intervention could be dependent on socioeconomic factors such as capacity to engage with the intervention, and appropriateness and acceptability of the intervention. Healthcare costs and quality of life related to obesity in childhood may also be dependent on SEP, but there is little published evidence that demonstrates this [17]. To make informed resource allocation decisions on interventions that also promote equity, for example, by targeting interventions to low socioeconomic groups, it is crucial to include economic evaluations demonstrating costs and health benefits across various socioeconomic groups [18].

This paper aimed to conduct a SEP-specific cost-effectiveness analysis of a telephone and short message service (SMS) intervention promoting healthy feeding practices, nutrition, physical activity and reducing screen time to reduce BMI growth in children aged 2–5 years in Sydney, Australia [19]. At 5 years of age, the intervention was shown to be more effective at reducing BMI growth in low rather than high SEP groups [19], suggesting that cost-effectiveness may also differ between the groups. To account for long-term impacts of obesity prevention beyond the duration of the trial, we undertook modelled cost-effectiveness analysis over a 12-year time horizon, for children from low- and high-SEP groups.

## Methods

### Study design and setting

We conducted a modelled economic evaluation of the telephone and SMS-based intervention compared to usual care, stratified by high and low SEP, from the perspective of the health care funder. This perspective was chosen because the intervention would most likely be implemented by a government agency. SEP was dichotomised for this evaluation to align with the sub-group analysis reported in the main trial [19]. The main trial study reported intervention effects on child BMI by annual household income above and below $80,000, which we used in our analysis as a proxy for high and low SEP. We modelled the differences in BMI found between intervention and usual care groups in the trial (ACTRN12618001571268) to estimate the incremental cost per BMI unit avoided and the incremental cost per quality-adjusted life year (QALY) gained using a 12-year time horizon. This time horizon was chosen to ensure the model captured the policy-relevant health and economic effects of the intervention. All costs were valued in 2023 Australian dollars and both costs and QALYs were discounted at 5% per year as recommended by Australian health funding guidelines [20, 21]. We used the Consolidated Health Economic Evaluation Reporting Standards (CHEERS; online Supporting Information) to adhere to best practice guidelines for the reporting of our analysis [22] (see Table S1).

### Intervention and comparator

The trial tested the effectiveness of a telephone and SMS-based intervention in reducing obesity risk of children aged 2–5 years. This was a 2-year extension of a RCT which used a three-staged telephone and SMS intervention to mothers of the same children aged 2–3 years [21]. The study was extended to support participating families during COVID-19. Study details of both the original and extended trials have been reported previously [19, 23].

In brief, there were five staged interventions conducted between 2 and 4 years of child age. Each intervention session included a mailout of educational materials, a 45–60-min telephone support call made by a nurse and 8 text messages over a 4-week period. The intervention was designed to support mothers regarding their child’s healthy eating, nutrition and physical activity with key messages focusing on feeding practices, active play and screen-viewing behaviours. The control group (usual care) did not receive any intervention. Children were followed up until 5 years of age, when child height and weight were measured by a research assistant for both groups.

The trial results were stratified into high and low household income as part of a subgroup analysis. Families were split into low- and high-income, based on an annual household income above and below $80,000. The household income for low-income families was below the Sydney median household income of $109,000 in 2020 [19]. For families with annual household income <$80,000, there was a difference in BMI at trial end of −0.57 (95% CI −1.05 to −0.10) units, whereas for high-income families (≥$80,000) there was a difference in BMI of −0.12 (95% CI −0.48 to 0.24) units [19].

### Intervention costs

The costs associated with the intervention were calculated as part of this economic evaluation and were determined by standard micro-costing techniques. They included the service provider costs and nurse time associated with telephone calls and SMS-support, administration time and educational materials. The length of each phone call was recorded, with the average length of time multiplied with the hourly wage of nurse educators as provided by public records in NSW, stratified by income group [24]. This allowed us to calculate resource use specific to each income group. Equipment and educational materials costs were estimated based on a previous micro-costing study of the earlier trial [25]. All costs are presented in AUD and where relevant, inflated to 2023 values using the latest Australian Health Price Index provided by the Australian Bureau of Statistics [26].

### EQuity-informative Early Prevention of Obesity in Childhood model

The EQuity-informative Early Prevention of Obesity in Childhood model (EQ-EPOCH) model is a discrete-time, microsimulation model developed and validated for conducting economic evaluations for childhood obesity prevention programs [15], that takes into account different child BMI growth among low- and high-SEP groups. In brief, BMI, health care costs, and quality-of life are computed over annual cycles, stratified by high and low SEP. In the model, SEP was defined as a composite measure accounting for parent education, parent occupation and family income [27]. SEP-specific BMI growth equations were developed using the kindergarten (K) cohort from the Longitudinal Study of Australian Children (LSAC), in which anthropometric and SEP measures were collected biennially from children aged 4 to 5 years until age 16 to 17 years. The model simulates faster BMI growth in the low-SEP group from 6 years of age and onwards, reflecting actual trends observed in Australian children and adolescents [28].

Direct health care costs and utility values are modelled based on weight status defined by World Health Organization (WHO) growth standards [29]. Weight status is categorised as healthy weight (including underweight) and overweight (including obesity). Healthcare costs by weight status were derived using a top-down approach using national Australian data (Supporting information) and utility values were derived from a meta-analysis [30]. The cost, utility and BMI change parameters that were included in the EQ-EPOCH model are presented in Table S2. Further details about the EQ-EPOCH model have been described elsewhere [15, 31].

### Model simulations

We assumed low and high household income as defined in the trial to be equivalent to low- and high-SEP, as defined by LSAC and in the EQ-EPOCH model. To incorporate uncertainty in the BMI effect sizes for the low- and high-SEP groups, we created 10,000 random draws of the SEP-specific BMI effect size found in the trial using a normal distribution (Table S2) and applied the BMI difference to a cohort of 4898 4- and 5-year-olds from the first wave of K cohort in LSAC. No SEP-specific cost or utility values were used, as a previous study found no differences by SEP-group. [17]. We ran the EQ-EPOCH model twice: the first time for a “control” cohort, where no intervention effects and costs were applied; and then for an “intervention” cohort, applying intervention effects and costs to predict individual BMI from 4/5 years until age 16/17 years. For both cohorts, sample weights provided in LSAC were applied. Incremental cost-effectiveness ratios (ICERs) were estimated separately for both low and high SEP groups. An incremental cost per BMI unit avoided and incremental cost per QALY gained was calculated by the difference in costs divided by the difference in BMI and QALYs among the intervention and control cohorts, simulated over 12 years.

### Uncertainty and sensitivity analyses

Deterministic one-way sensitivity analyses were conducted using the upper and lower 95% confidence intervals for the SEP-specific parameters: intervention effect size and intervention costs. We used one standard deviation away from the mean of the utility values associated with overweight or obesity in sensitivity analyses and also used discount rates of 3% and 7% and ±25% of direct health care costs.

To estimate joint uncertainty in both costs and QALYs, we took 10,000 bootstrapped samples with replacement of the LSAC cohort by SEP and plotted the 10,000 bootstrapped cost and QALY pairs on an incremental cost-effectiveness plane. The bootstrapped samples were also used to estimate the probability the intervention would be cost-effective at a threshold of $50,000/QALY, an unofficial threshold in healthcare decision-making in Australia [32].

All analyses were conducted in StataBE 18.0.

## Results

A summary of the intervention costs per child, stratified by SEP are shown in Table S3. The high SEP group, overall, spent on average 175 (95% CI 158–192) min on the telephone service, slightly higher than the low SEP group of 145 (95% CI 123–167) min. These differences were due to a higher proportion of mothers engaging with the telephone service in the high-SEP (90.3%) compared to the low-SEP (76.6%) group. The mean costs per child associated with the telephone service were higher for the high- ($218) compared to the low-SEP ($181) group. Overall, the intervention costs were $219 (95% CI 191–246) and $256 (95% CI 235–277) per child in the low- and high-SEP groups, respectively.

The distribution of characteristics at the end of follow-up for the trial and LSAC K cohorts are presented in Table 1. The mean BMI in the low- and high-SEP groups in the control arm of the trial were similar to the respective LSAC cohorts. The trial had slightly more males in the low SEP group and slightly more females in the high SEP group compared to the LSAC counterparts.

**Table 1:** Characteristics of participants in trial and simulation input cohorts at from ages 3–5 years.

	Intervention	Control	Intervention	Control	Low SEP	High SEP
	Low SEP		High SEP		LSAC K cohort	
Sample size	105	111	196	189	2683	2215
Age, mean (SD)	5.05 (0.15)	5.05 (0.13)	5.04 (0.11)	5.04 (0.10)	4.79 (0.22)	4.78 (0.22)
BMI^a^, mean (SD)	15.76 (0.14)	16.34 (0.12)	15.99 (0.10)	16.11 (0.10)	16.40 (1.68)	16.18 (1.52)
Male, n(%)	60 (57.14%)	58 (52.25%)	96 (48.98%)	89 (47.09%)	1360 (50.69%)	1126 (50.84%)

*CHAT* Communicating Healthy Beginning Advice by Telephone, *SEP* socioeconomic position, *BMI* body-mass index, *LSAC K cohort* Longitudinal Study of Australian Children Kindergarten cohort, *SD* Standard Deviation.

^a^Mean BMI for both low- and high-SEP groups was calculated using longitudinal and intention-to-treat analysis with multiple imputation (sample size was 662 children).

**Fig. 1:**
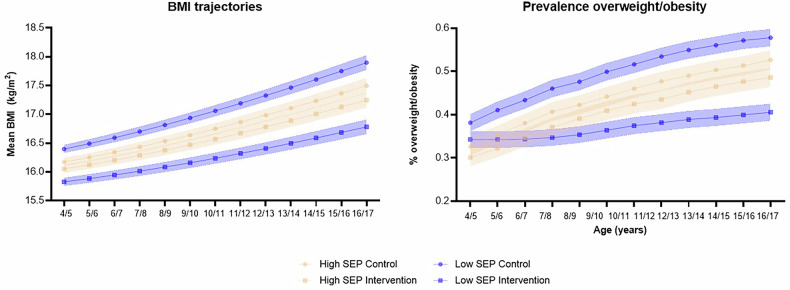
Modelled BMI trajectories (95% CI) for the low- and high-SEP groups, by control and intervention group, using the EQuity-informative Early Prevention of Obesity in Childhood (EQ-EPOCH) model. BMI body mass index, SEP socioeconomic position.

The modelled BMI trajectories over a 12-year timeframe are shown in Fig. 1. Applying the intervention effect size at age 4/5 years saw both groups move onto a lower BMI trajectory and resulted in a reduction of 1.12 (95% 0.94–1.29) BMI units in the low-SEP group compared to a 0.25 (95% CI 0.07 to −0.43) BMI unit reduction in the high-SEP group at age 16/17. This translates to a reduction in the prevalence of overweight and obesity by 17.03% (95% CI 14.40–19.67) and 4.06% (95% 0.01–0.07) in the low- and high-SEP groups, respectively. Importantly the inequality in BMI reversed over time, as mean BMI in adolescence for the low-SEP group under intervention was predicted to be 0.46 (95% CI 0.29–0.64) BMI units lower than the high-SEP group.

**Fig. 2:**
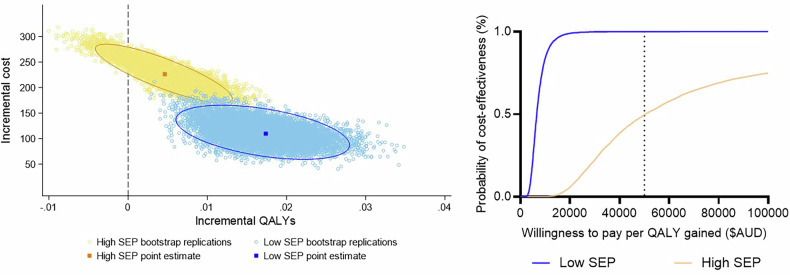
Cost-effectiveness results by socioeconomic position. **Left Panel:** Cost-effectiveness plane presenting incremental costs and QALYs for each bootstrapped sample for each SEP group. **Right Panel:** Cost-effectiveness acceptability curves presenting the probability of cost-effectiveness under a range of willingness to pay thresholds. AUD Australian dollar, QALY quality-adjusted life year, SEP socioeconomic position.

The incremental costs were smaller in the low-SEP group ($112) when compared to the high-SEP group ($227), which was driven by offsets in health care costs associated with a greater reduction in overweight and obesity (Table 2). Incremental BMI and incremental QALYs were greater in the low-SEP group, and this meant the ICERs were cost-effective in this group ($131 per BMI unit avoided/$6 549 per QALY gained). The high-SEP group was marginally cost-effective, with higher ICERs for both cost-effectiveness and cost-utility at $1161 per BMI unit avoided and $41,462 per QALY gained. When accounting for joint uncertainty in costs and QALYs, 90.0% of the bootstrapped samples in the high-SEP group lay within the north-east quadrant of the cost-effectiveness plane (Fig. 2), indicating that the intervention incurred more healthcare costs, but was more effective than usual care. In the low-SEP group 100% of the bootstrapped samples lay in the north-east quadrant, indicating that the intervention was more costly and more effective than usual care. At the $50,000 per QALY gained threshold, the intervention had a 99.97% probability of being cost-effective in the low-SEP group and a 49.63% probability in the high-SEP group (Table 2). This indicates that the intervention is extremely likely to be cost-effective for the low-SEP group, but marginally cost-effective in the high-SEP group.

**Table 2:** Incremental costs, effects and QALYs.

	Low SEP	High SEP
Incremental costs, mean (bootstrapped 95% CI)	$112.37 (111.95–112.79)	$227.41 (226.95–227.87)
Incremental BMI at age 16/17 years (bootstrapped 95% CI)	0.84 (0.84–0.85)	0.16 (0.15–0.17)
Incremental QALYs (bootstrapped 95% CI)	0.0170 (0.0169–0.0171)	0.0045 (0.0044–0.0046)
ICER (A$ per unit BMI avoided)	$131 (73–265)	$1161 (−5549 to 8076)
ICER (A$ per QALY gained)	$6549 (3607–13,953)	$41,462 (−2174 to 85,097)
Probability cost-effective at $50,000/QALY threshold	99.97%	49.63%

All costs and outcomes presented with 95% confidence intervals of 10,000 bootstrap replications. Costs and QALYs discounted at 5%.

*BMI* body mass index, *QALY* quality-adjusted life years, *SEP* socioeconomic position.

**Fig. 3:**
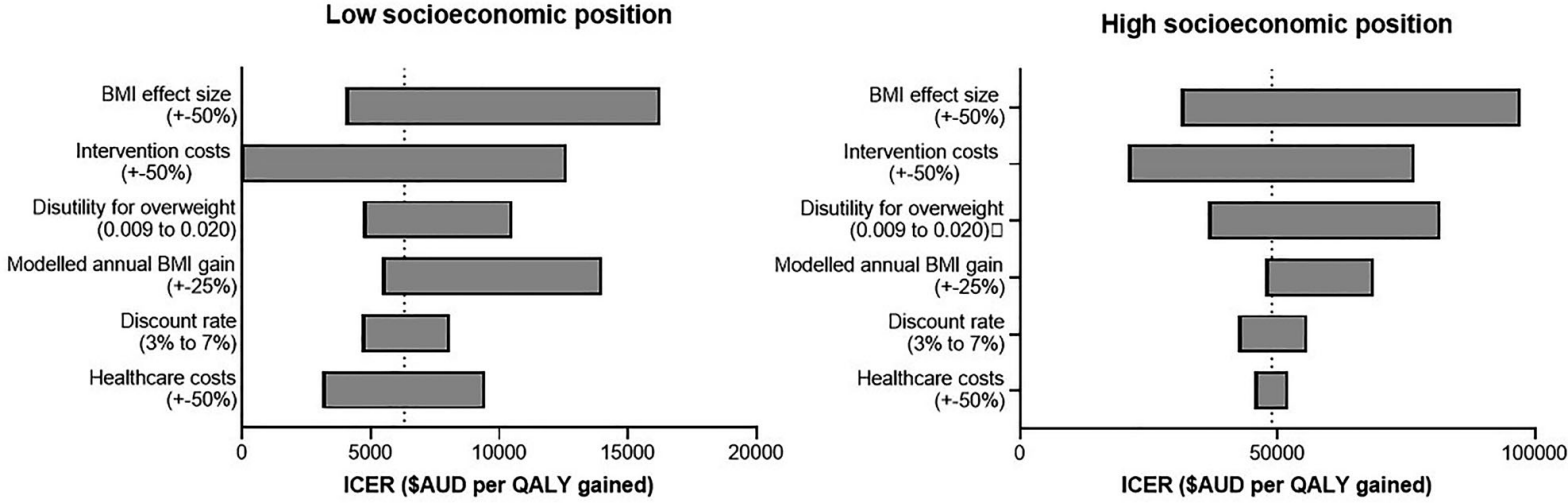
Sensitivity analyses. Tornado plots representing the range of cost-utility ICERs using alternative analysis parameters. AUD Australian dollar, ICER Incremental cost-effectiveness ratio, QALY quality-adjusted life year, SEP socioeconomic position.

One-way sensitivity analyses are presented in Fig. 3 and Table S4. The ICERs were most sensitive to changes in the intervention effect size, intervention costs, modelled annual BMI gain and for the high-SEP group only, the disutility for overweight. ICERs were minimally sensitive to changes in the discount rate, the disutility of overweight (low-SEP group only) and healthcare costs (high-SEP group only). In all sensitivity analyses conducted, the overall finding that the intervention was more cost-effective in the low-SEP group compared to the high-SEP group did not change.

## Discussion

The study showed that a health promotion intervention designed to prevent early childhood obesity, implemented through telephone and SMS, was cost-effective for the low-SEP group, but not necessarily for the high-SEP group. We used the EQ-EPOCH model, a fit-for-purpose health economic model that has been previously validated and accurately predicts BMI trajectories by low- and high-SEP groups in Australia. When accounting for uncertainty, the intervention had a very high probability in the low-SEP group and a moderate probability in the high-SEP group of being cost-effective at the $50,000 per QALY gained threshold commonly used in Australia [32].

Our study is, to the best of our knowledge, the first SEP-specific economic evaluation of a recently conducted early childhood obesity prevention intervention conducted in Australia. One previous SEP-specific economic evaluation of early childhood obesity interventions has been published [15], using SEP-specific effect sizes from the Prevention of Overweight in Infancy (POI) trial [33]. The study used the EQ-EPOCH model to predict greater cost-effectiveness in low- and mid-SEP groups when compared to high-SEP groups [15]. However, several assumptions were made for these studies. Firstly, the effectiveness results came from a trial that was not powered to detect effects within SEP groups and was conducted over 12 years ago. Secondly, the trial was conducted in New Zealand with the assumption that a similar effect size across different SEP groups could be achieved if the intervention was transported to the Australian context. By contrast, the effectiveness data for our study was derived from an RCT that was conducted in Australia, thereby eliminating the need for assumptions regarding the transportability of effectiveness to another country.

The differential cost-effectiveness across the SEP groups was largely driven by the greater reduction in BMI for the low-SEP group found in the trial. Even though the low-SEP group had a higher BMI than the high-SEP group before the intervention effect was applied, the magnitude of their BMI reduction was substantial enough to shift them onto a new trajectory (see Fig. 1). By ages 16–17 years, this resulted in a lower average BMI compared to the high-SEP group, resulting in greater cost-effectiveness and improved equity. The one-way sensitivity analyses showed that the results were most sensitive to uncertainty in the intervention effect size. This is unsurprising given the relatively large BMI reduction found in the trial [19]. However, it is worth noting that all the sensitivity analyses conducted did not change the conclusion that the intervention was more cost-effective for the low-SEP group.

Our study has important implications on how best to implement this intervention for decision-makers. This is a telehealth intervention that has the potential to be scaled up at a state or federal level. The intervention (telephone and SMS support and mailed educational materials) offers significant advantages over delivering the intervention face-to-face. This approach could facilitate access for children residing in rural and remote regions, where challenges such as staff shortages and extensive travel times can hinder service delivery.

Given that the intervention is highly cost-effective in the low SEP group and marginally cost-effective in the high SEP group, focusing implementation efforts in areas with a greater concentration of individuals from low SEP backgrounds would simultaneously ensure value for money and promote equity. An example would be to target areas using the Socio-Economic Indexes for Areas (SEIFA) in Australia, which ranks areas according to their relative socio-economic advantage and disadvantage using national Census data [34]. However, to maximise the potential benefits of the intervention, efforts must focus on increasing engagement with the telephone and SMS support intervention for participants from low-SEP backgrounds, as evidence from this trial and a previous study indicates greater engagement in high-SEP groups [35]. Greater engagement will increase the average cost of the intervention in the low-SEP group but potentially improve the cost-effectiveness if implemented at scale.

A major strength of this analysis is the use of the EQ-EPOCH model, a validated, BMI trajectory model that predicts individual-level BMI by SEP. A recent review showed a dearth of cost-effectiveness modelling studies that used separate BMI trajectories by SEP [36]. The microsimulation model is well suited to account for heterogeneity in the BMI distribution and model health outcomes at an individual-level. Our analysis used a large cohort (LSAC K cohort) of 4898 Australian children aged 4/5 years with individual-level data on age, sex, BMI and SEP. The parameters of the model were informed by the best available evidence, including a systematic review and meta-analysis of utility decrements by weight status [30], national level data on health care costs [26] and RCT evidence for intervention costs and change in BMI [19].

However, our study is not without its limitations. Collected in 2004, the LSAC data may not reflect the present BMI and sociodemographic profiles of children aged 4 to 5 years. Although the proportion of children with overweight or obesity has remained relatively stable between 2007–2008 (23%) and 2017–2018 (24%) [36], the relative proportions between high and low SEP groups may have changed. Further work is needed to determine the generalisability of our findings to the current context. We have assumed the definition of low- and high-SEP in the trial (above and below household income of $80,000) to be equivalent to the definition of SEP in the EQ-EPOCH model, which uses a composite measure of SEP including household income but also encompassing parents’ education and occupation [27]. We have also assumed SEP to be constant over a 12-year period, which may impact BMI trajectories for children that experience changes in their SEP over time. Finally, as the primary outcomes analysis of the trial from which the inputs of this economic evaluation were derived [19] only included a subgroup analysis above and below a household income of $80,000, more complex analyses such as other SEP categorisations and effects by ethnicity or cultural diversity could not be incorporated. This means that, despite evidence that some cultural and linguistically diverse groups in Australia have higher risk of obesity [37], the intersectionality of cultural diversity and SEP could not be examined in this study.

## Conclusions

Our study shows compelling economic evidence to support decision makers to address health inequities in early childhood. We have demonstrated that a telephone and SMS based health promotion intervention for mothers with children aged 2–4 years is highly cost-effective in the low-SEP group, one of the priority populations who have the highest rates of overweight and obesity in Australia. Targeting this intervention to areas where families from low-SEP backgrounds are overrepresented would represent good value for money, as well as improving equity. Future work should consider how implementation factors, such as reach, uptake, and economies of scale [38] might affect the cost-effectiveness of the intervention across different socioeconomic groups.

## Supplementary information

Supplementary Information

## Data Availability

The Longitudinal Study of Australian Children data are available from the Longitudinal Studies Dataverse website (https://dataverse.ada.edu.au/dataverse/lsac) for those who meet the criteria for access to de-identified LSAC data.

## Code availability

The STATA code used for this study is available upon request to the corresponding author.

## References

[1] World Health Organisation. Report of the Commission On Ending Childhood Obesity. WHO; Geneva, Switzerland 2016. https://www.who.int/publications/i/item/9789241510066

[2] Australian Institute of Health and Welfare. Overweight and obesity. 2022. https://www.aihw.gov.au/reports/australias-health/overweight-and-obesity.

[3] Griffiths LJ, Parsons TJ, Hill AJ. Self-esteem and quality of life in obese children and adolescents: a systematic review. Int J Pediatr Obes. 2010;5:282–304. 20210677 10.3109/17477160903473697

[4] Hayes A, Chevalier A, D’Souza M, Baur L, Wen LM, Simpson J. Early childhood obesity: association with healthcare expenditure in Australia. Obesity. 2016;24:1752–8. 27380909 10.1002/oby.21544

[5] Hayes AJ, Brown V, Tan EJ, Chevalier A, D’Souza M, Rissel C, et al. Patterns and costs of health-care utilisation in Australian children: the first 5 years. J Paediatr Child Health. 2019;55:802–8. 30411424 10.1111/jpc.14292

[6] Carrello J, Lung T, Killedar A, Baur LA, Hayes A. Relationship between obesity and school absenteeism in Australian children: implications for carer productivity. Obes Res Clin Pract. 2021;15:587–92. 34625400 10.1016/j.orcp.2021.09.006

[7] Hayes AJ, Carrello JP, Kelly PJ, Killedar A, Baur LA. Looking backwards and forwards: tracking and persistence of weight status between early childhood and adolescence. Int J Obes. 2021;45:870–8. 10.1038/s41366-021-00751-3 33558641

[8] Sankar UV, Kutty VR, Anand TN. Measuring childhood socioeconomic position in health research: development and validation of childhood socioeconomic position questionnaire using mixed method approach. Health Promot Perspect. 2019;9:40–9. 30788266 10.15171/hpp.2019.05 PMC6377695

[9] Chung A, Backholer K, Wong E, Palermo C, Keating C, Peeters A. Trends in child and adolescent obesity prevalence in economically advanced countries according to socioeconomic position: a systematic review. Obes Rev. 2016;17:276–95. 26693831 10.1111/obr.12360

[10] Johnson LG, Cho H, Lawrence SM, Keenan GM. Early childhood (1-5 years) obesity prevention: a systematic review of family-based multicomponent behavioral interventions. Prev Med. 2024;181:107918. 38417469 10.1016/j.ypmed.2024.107918

[11] Brown T, Moore TH, Hooper L, Gao Y, Zayegh A, Ijaz S, et al. Interventions for preventing obesity in children. Cochrane Database Syst Rev. 2019;7:CD001871. 31332776 10.1002/14651858.CD001871.pub4 PMC6646867

[12] Venturelli F, Ferrari F, Broccoli S, Bonvicini L, Mancuso P, Bargellini A, et al. The effect of Public Health/Pediatric Obesity interventions on socioeconomic inequalities in childhood obesity: a scoping review. Obes Rev. 2019;20:1720–39. 10.1111/obr.12931. 31468647 10.1111/obr.12931 PMC6899709

[13] Beauchamp A, Backholer K, Magliano D, Peeters A. The effect of obesity prevention interventions according to socioeconomic position: a systematic review. Obes Rev. 2014;15:541–54. 24629126 10.1111/obr.12161

[14] Drummond MF, Sculpher MJ, Claxton K, Stoddart G, Torrance G. Methods for the economic evaluation of health care programmes. Oxford: Oxford University Press; 2015.

[15] Killedar A, Lung T, Taylor RW, Taylor BJ, Hayes A. Is the cost-effectiveness of an early-childhood sleep intervention to prevent obesity affected by socioeconomic position?. Obesity. 2023;31:192–202. 36471911 10.1002/oby.23592 PMC10947595

[16] Killedar A, Lung T, Taylor RW, Hayes A. Modelled distributional cost-effectiveness analysis of childhood obesity interventions: a demonstration. Appl Health Econ Health Policy. 2023;21:615–25. 10.1007/s40258-023-00813-9. 37221341 10.1007/s40258-023-00813-9 PMC10232580

[17] Killedar A, Lung T, Petrou S, Teixeira-Pinto A, Tan EJ, Hayes A. Weight status and health-related quality of life during childhood and adolescence: effects of age and socioeconomic position. Int J Obes. 2020;44:637–45. 10.1038/s41366-020-0529-3 31949296

[18] Espinoza MA, Manca A, Claxton K, Sculpher MJ. The value of heterogeneity for cost-effectiveness subgroup analysis: conceptual framework and application. Med Decis Making. 2014;34:951–64. 24944196 10.1177/0272989X14538705 PMC4232328

[19] Wen LM, Xu H, Chen Z, Hayes A, Phongsavan P, Taki S, et al. Effectiveness of a telephone-based randomised clinical trial targeting obesity risk of preschool-aged children: An extension study during the COVID-19 pandemic. Int J Obes. 2025. 10.1038/s41366-025-01869-4. 10.1038/s41366-025-01869-4 PMC12583190 40813489

[20] Australian Department of Health. Guidelines for preparing submissions to the Pharmaceutical Benefits Advisory Committee (PBAC). 2016. https://pbac.pbs.gov.au/.

[21] NSW Treasury. TPG23-08 NSW Government guide to cost-benefit analysis. 2023. https://www.treasury.nsw.gov.au/sites/default/files/2023-04/tpg23-08_nsw-government-guide-to-cost-benefit-analysis_202304.pdf.

[22] Husereau D, Drummond M, Augustovski F, de Bekker-Grob E, Briggs AH, Carswell C, et al. CHEERS 2022 ISPOR Good Research Practices Task Force. Consolidated Health Economic Evaluation Reporting Standards 2022 (CHEERS 2022) Statement: updated reporting guidance for health economic evaluations. Value Health. 2022;25:3–9. 35031096 10.1016/j.jval.2021.11.1351

[23] Wen LM, Xu H, Phongsavan P, Rissel C, Hayes A, Taki S, et al. Twelve-month effectiveness of telephone and SMS support to mothers with children aged 2 years in reducing children’s BMI: a randomized controlled trial. Int J Obes. 2023;47:791–8. 10.1038/s41366-023-01311-7 PMC10121422 37087468

[24] NSW Ministry of Health. Public health system nurses’ and midwives’ (State) award 2023. Industrial relations commission of New South Wales. 2024. https://www.health.nsw.gov.au/careers/conditions/awards/nurses.pdf.

[25] Brown V, Tan EJ, Hayes A, Baur L, Campbell K, Taylor R, et al. Cost comparison of five Australasian obesity prevention interventions for children aged from birth to two years. Pediatr Obes. 2020;15:e12684. 32558343 10.1111/ijpo.12684

[26] Australian Bureau of Statistics. Consumer Price Index, Australia. 2024. https://www.abs.gov.au/statistics/economy/price-indexes-and-inflation/consumer-price-index-australia/latest-release.

[27] Baker K, Sipthorp M, Edwards B. A longitudinal measure of socioeconomic position in LSAC. Australian Institute of Family Studies; 2017.

[28] Killedar A, Lung T, Hayes A. Investigating socioeconomic inequalities in BMI growth rates during childhood and adolescence. Obes Sci Pract. 2021;8:101–11. 35127126 10.1002/osp4.549 PMC8804938

[29] World Health Organizatioo. Growth reference data for 5-19 years. 2024. https://www.who.int/tools/growthreference-data-for-5to19-years.

[30] Brown V, Tan EJ, Hayes AJ, Petrou S, Moodie ML. Utility values for childhood obesity interventions: a systematic review and meta-analysis of the evidence for use in economic evaluation. Obes Rev. 2018;19:905–16. 29356315 10.1111/obr.12672

[31] Killedar AA. Modelling the cost-effectiveness and equity impact of strategies to prevent childhood obesity in Australia. Thesis. The University of Sydney; 2021. https://ses.library.usyd.edu.au/handle/2123/27413.

[32] Wang S, Gum D, Merlin T. Comparing the ICERs in medicine reimbursement submissions to NICE and PBAC-does the presence of an explicit threshold affect the ICER proposed?. Value Health. 2018;21:938–43. 30098671 10.1016/j.jval.2018.01.017

[33] Taylor BJ, Gray AR, Galland BC, Heath AM, Lawrence J, Sayers RM, et al. Targeting sleep, food, and activity in infants for obesity prevention: an RCT. Pediatrics. 2017;139:e20162037. 28242860 10.1542/peds.2016-2037

[34] Australian Bureau of Statistics. Technical Paper Socio-Economic Indexes for Areas (SEIFA) 2016 Catalogue No. 2033.0.55.001. 2024. https://www.abs.gov.au/ausstats/abs@.nsf/Lookup/2033.0.55.001main+features12016.

[35] Ekambareshwar M, Xu H, Rissel C, Baur L, Taki S, Mihrshahi S, et al. Participants’ engagement with telephone support interventions to promote healthy feeding practices and obesity-protective behaviours for infant obesity prevention. Front Endocrinol. 2022;13:868944. 10.3389/fendo.2022.868944 PMC9108251 35586630

[36] Onyimadu O, Violato M, Astbury NM, Hüls H, Heath L, Shipley A, et al. A systematic review of economic evaluations of interventions targeting childhood overweight and obesity. Obes Rev. 2023;24:e13597. 37463862 10.1111/obr.13597

[37] Lung T, Killedar A, Taki S, Wen LM, Dickson M, Howard K, et al. Differences in weight status among Australian children and adolescents from priority populations: a longitudinal study. Int J Obes. 2024;48:702–8. 10.1038/s41366-024-01471-0 PMC11058044 38307955

[38] Dalton C, Sÿultana M, McKenna K, Brown V. How is scale incorporated into the economic evaluation of interventions to prevent obesity or to improve obesity-related risk factors: a systematic scoping review. Obes Rev. 2025;26:e13942. 40400024 10.1111/obr.13942 PMC12318913

