# Supplementary material for: Socioeconomic differences in the cost-effectiveness of a telephone-based intervention for obesity prevention in early childhood
Source: Int J Obes (Lond). 2025 Sep 6;50(1):152–8. doi: 10.1038/s41366-025-01904-4 (PMC12855013; doi:10.1038/s41366-025-01904-4)
Supplement: Supplementary file 1 — Supplementary Information [file 41366_2025_1904_MOESM1_ESM.docx]

**Supplementary Information**

**Table S1** - CHEERS 2022 Checklist

| **Topic** | **No.** | **Item** | **Location where item is reported** |
| --- | --- | --- | --- |
| **Title** |  |  |  |
|  | 1 | Identify the study as an economic evaluation and specify the interventions being compared. | 1 |
| **Abstract** |  |  |  |
|  | 2 | Provide a structured summary that highlights context, key methods, results, and alternative analyses. | 2 |
| **Introduction** |  |  |  |
| **Background and objectives** | 3 | Give the context for the study, the study question, and its practical relevance for decision making in policy or practice. | 3-4 |
| **Methods** |  |  |  |
| **Health economic analysis plan** | 4 | Indicate whether a health economic analysis plan was developed and where available. | NA |
| **Study population** | 5 | Describe characteristics of the study population (such as age range, demographics, socioeconomic, or clinical characteristics). | 4-7, Table 1 |
| **Setting and location** | 6 | Provide relevant contextual information that may influence findings. | 4-5 |
| **Comparators** | 7 | Describe the interventions or strategies being compared and why chosen. | 4-7 |
| **Perspective** | 8 | State the perspective(s) adopted by the study and why chosen. | 4 |
| **Time horizon** | 9 | State the time horizon for the study and why appropriate. | 7 |
| **Discount rate** | 10 | Report the discount rate(s) and reason chosen. | 7 |
| **Selection of outcomes** | 11 | Describe what outcomes were used as the measure(s) of benefit(s) and harm(s). | 7 |
| **Measurement of outcomes** | 12 | Describe how outcomes used to capture benefit(s) and harm(s) were measured. | 7 |
| **Valuation of outcomes** | 13 | Describe the population and methods used to measure and value outcomes. | 6-7 |
| **Measurement and valuation of resources and costs** | 14 | Describe how costs were valued. | 4-6 |
| **Currency, price date, and conversion** | 15 | Report the dates of the estimated resource quantities and unit costs, plus the currency and year of conversion. | 4 |
| **Rationale and description of model** | 16 | If modelling is used, describe in detail and why used. Report if the model is publicly available and where it can be accessed. | 6-7 |
| **Analytics and assumptions** | 17 | Describe any methods for analysing or statistically transforming data, any extrapolation methods, and approaches for validating any model used. | 6-7, see reference 14 |
| **Characterising heterogeneity** | 18 | Describe any methods used for estimating how the results of the study vary for subgroups. | Whole manuscript |
| **Characterising distributional effects** | 19 | Describe how impacts are distributed across different individuals or adjustments made to reflect priority populations. | 5-7 |
| **Characterising uncertainty** | 20 | Describe methods to characterise any sources of uncertainty in the analysis. | 7 |
| **Approach to engagement with patients and others affected by the study** | 21 | Describe any approaches to engage patients or service recipients, the general public, communities, or stakeholders (such as clinicians or payers) in the design of the study. | NA |
| **Results** |  |  |  |
| **Study parameters** | 22 | Report all analytic inputs (such as values, ranges, references) including uncertainty or distributional assumptions. | 8-9 |
| **Summary of main results** | 23 | Report the mean values for the main categories of costs and outcomes of interest and summarise them in the most appropriate overall measure. | 8 |
| **Effect of uncertainty** | 24 | Describe how uncertainty about analytic judgments, inputs, or projections affect findings. Report the effect of choice of discount rate and time horizon, if applicable. | 8-9 |
| **Effect of engagement with patients and others affected by the study** | 25 | Report on any difference patient/service recipient, general public, community, or stakeholder involvement made to the approach or findings of the study | NA |
| **Discussion** |  |  |  |
| **Study findings, limitations, generalisability, and current knowledge** | 26 | Report key findings, limitations, ethical or equity considerations not captured, and how these could affect patients, policy, or practice. | 9-12 |
| **Other relevant information** |  |  |  |
| **Source of funding** | 27 | Describe how the study was funded and any role of the funder in the identification, design, conduct, and reporting of the analysis | 13 |
| **Conflicts of interest** | 28 | Report authors conflicts of interest according to journal or International Committee of Medical Journal Editors requirements. | 13 |

*From:* Husereau D, Drummond M, Augustovski F, et al. Consolidated Health Economic Evaluation Reporting Standards 2022 (CHEERS 2022) Explanation and Elaboration: A Report of the ISPOR CHEERS II Good Practices Task Force. Value Health 2022;25. <doi:10.1016/j.jval.2021.10.008>

**Table S2** - Parameters used in the economic evaluation

| Parameters | Values | Source |
| --- | --- | --- |
| BMI growth equations | Used the EQ-EPOCH model BMI growth equations accounting for current BMI, age, SEP and stratified by sex | Killedar et al. 2023 (1) |
| Intervention costs | \| Low SEP \| $219 (191 – 246) \| \| --- \| --- \| \| High SEP \| $256 (235 – 280) \| | Trial data and Brown et al. 2020 (2) |
| Healthcare costs associated with weight status | \| Age \| 5-9 \| 10-14 \| 15-19 \| \| --- \| --- \| --- \| --- \| \| Healthy \| $805 \| $863 \| $1391 \| \| Overweight \| $909 \| $975 \| $1571 \| | Killedar et al. 2023 (1) and inflated to 2023 values (3) |
| Utility values | \| Healthy \| 0.85 \| \| --- \| --- \| \| Disutility for overweight \| 0.015 (0.003 to 0.026) \| | Brown et al. 2018 (4) |
| Intervention effect size (change in BMI) | \| Low SEP \| -0.57 (-1.05 to -0.10) \| \| --- \| --- \| \| High SEP \| -0.12 (-0.48 to 0.24) \| | Wen et al. 2024 (5) |

BMI: Body-mass index; SEP: Socioeconomic position
The probabilistic sensitivity analysis randomly sampled values for the parameters presented with 95% confidence intervals using normal distributions

References

1. Killedar A, Lung T, Taylor RW, Taylor BJ, Hayes A. Is the cost-effectiveness of an early-childhood sleep intervention to prevent obesity affected by socioeconomic position? Obesity (Silver Spring). 2023 Jan;31(1):192-202
2. Brown V, Tan EJ, Hayes A, Baur L, Campbell K, Taylor R, Byrne R, Wen LM, Hesketh KD, Moodie M. Cost comparison of five Australasian obesity prevention interventions for children aged from birth to two years. Pediatr Obes. 2020 Dec;15(12):e12684.
3. Australian Bureau of Statistics. Consumer Price Index, Australia. Accessed 8 August, 2024. https://www.abs.gov.au/statistics/economy/price-indexes-and-inflation/consumer-price-index-australia/latest-release
4. Brown V, Tan EJ, Hayes AJ, Petrou S, Moodie ML. Utility values for childhood obesity interventions: a systematic review and meta-analysis of the evidence for use in economic evaluation. Obes Rev. 2018 Jul;19(7):905-916.
5. Wen LM, Xu H, Chen Z, Hayes A, Phongsavan P, Taki S, Kerr E, Jawad D, Simone L, Rissel C, Baur L., Effectiveness of a Telephone-Based Randomised Controlled Trial Targeting Obesity Risk of Preschool-Aged Children: An Extension Study During the COVID-19 Pandemic. Available at SSRN: https://ssrn.com/abstract=4875995

**Table S3** – Intervention costs

| Intervention costs per child | Low SEP  Mean (95% CI) | High SEP  Mean (95% CI) |
| --- | --- | --- |
| Equipment and educational material costs | $38 (37-39) | $38 (37-39) |
| Nurse consultation time | $181 (154 – 209) | $218 (197 – 239) |
| **Total cost** | $219 (191 – 246) | $256 (235 – 280) |

**Table S4** – Description and presentation of sensitivity analyses

| Parameter | Description | Low SEP | | High SEP | |
| --- | --- | --- | --- | --- | --- |
|  |  | CEA ICER | CUA ICER | CEA ICER | CUA ICER |
| Base case |  | $126 | $6307 | $1426 | $48 911 |
| Utilities | Lower bounds (LB) and upper bounds (UB) of 1 standard deviation away from the mean disutility of overweight were used (See Appendix Table 2) | NA | \| LB: 14001 \| \| --- \| \| UB: 5488 \| | NA | \| LB: 81526 \| \| --- \| \| UB: 36687 \| |
| Healthcare costs | LB and UB of healthcare costs were 50% lower and higher than estimated in the base case | \| LB: 194 \| \| --- \| \| UB: 65 \| | \| LB: 9445 \| \| --- \| \| UB: 3170 \| | \| LB: 1519 \| \| --- \| \| UB: 1332 \| | \| LB: 52109 \| \| --- \| \| UB: 45712 \| |
| Intervention costs | LB and UB of intervention costs were 50% lower and higher than estimated in the trial, by SEP | \| LB: 0  UB:258 \| \| --- \| | \| LB: 16  UB:12598 \| \| --- \| | \| LB: 620 \| \| --- \| \| UB: 2232 \| | \| LB: \| \| --- \| \| UB: 76564 \| |
| Intervention effect size | LB and UB of the change in BMI taken from the trial being 50% lower and higher than estimated in the trial, by SEP | \| LB: 363 \| \| --- \| \| UB: 69 \| | \| LB: 16265 \| \| --- \| \| UB: 4047 \| | \| LB: 3005 \| \| --- \| \| UB: 898 \| | \| LB: 97271 \| \| --- \| \| UB: 31609 \| |
| Annual BMI gain | Tested the effect of BMI gain being 25% lower and higher than that modelled by EQ-EPOCH at every annual cycle | \| LB: 198 \| \| --- \| \| UB: 110 \| | \| LB: 14001 \| \| --- \| \| UB: 5488 \| | \| LB: 1584  UB: 1334 \| \| --- \| | \| LB: 68649 \| \| --- \| \| UB: 47972 \| |
| Discount rate | We tested the effect of changing the discount rate for costs and QALYs from 5% in the base case analysis to 3% and 7% | \| LB: 110 \| \| --- \| \| UB: 145 \| | \| LB: 4676 \| \| --- \| \| UB: 8085 \| | \| LB: 1401  UB: 1446 \| \| --- \| | \| LB: 42510 \| \| --- \| \| UB: 55748 \| |

CEA: Cost-effectiveness analysis, CUA: Cost-utility analysis, ICER: Incremental cost-effectiveness ratio, QALYs: Quality-adjusted life years, SEP: Socioeconomic position

CEA ICERs are presented as $AUD per BMI unit avoided and CUA ICERs as $AUD per QALY gained

**Figure S1** – Cost-effectiveness plane presenting incremental costs and body-mass index for each bootstrapped sample for each SEP group

BMI: body-mass index; SEP: socioeconomic position

**Figure S2** - Sensitivity analyses. Tornado plots representing the range of cost-utility ICERs using alternative analysis parameters.


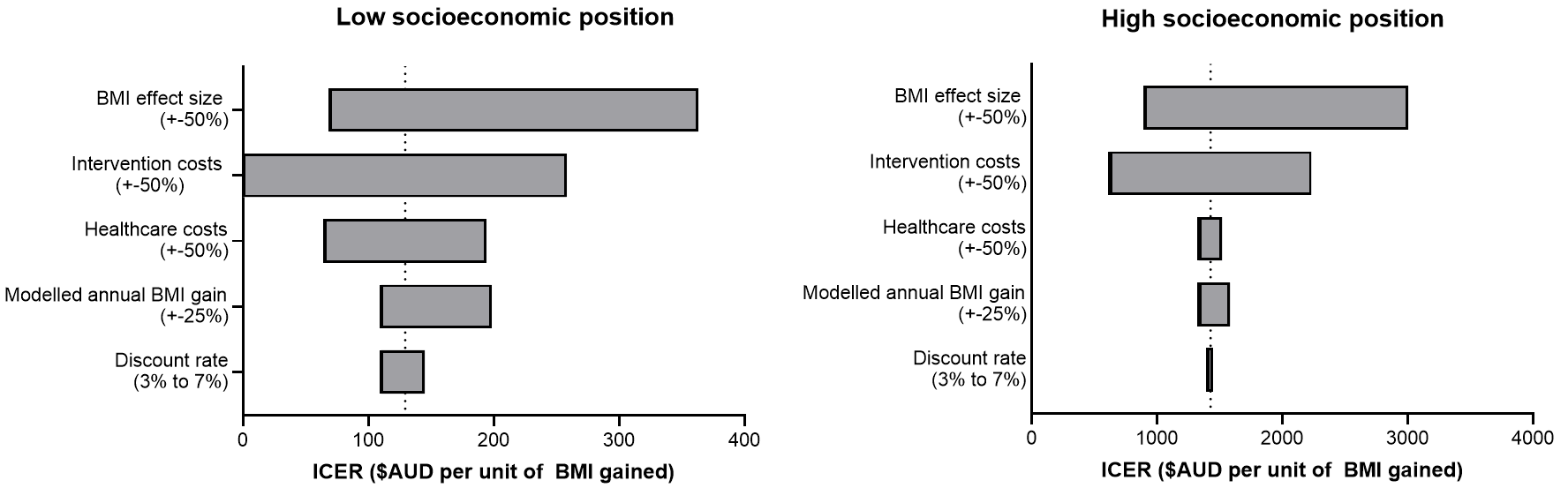

AUD: Australian dollar; ICER: Incremental cost-effectiveness ratio; QALY: quality-adjusted life year; SEP, socioeconomic position
